# Neurodegeneration in the retina of motoneuron diseases: a longitudinal study in amyotrophic lateral sclerosis and Kennedy’s disease

**DOI:** 10.1007/s00415-023-11802-2

**Published:** 2023-06-08

**Authors:** Alessandro Miscioscia, Marco Puthenparampil, Lorenzo Blasi, Francesca Rinaldi, Paola Perini, Gianni Sorarù, Paolo Gallo

**Affiliations:** 1 Department of Neurosciences, DNS, School of Medicine, University of Padua, Padua, Italy; 2 Multiple Sclerosis Centre of the Veneto Region (CeSMuV), Neurology Clinic, University Hospital of Padua, Via Giustiniani, 5, 35128 Padua, Italy; 3 Neuromuscular Center, Neurology Clinic, University Hospital of Padua, Padua, Italy

**Keywords:** Optical coherence tomography, Amyotrophic lateral sclerosis, Kennedy’s disease, Motoneuron disease, Neurodegeneration, Biomarker

## Abstract

**Background:**

To what extent retinal atrophy in neurodegenerative diseases reflects the severity and/or the chronicity of brain pathology or is a local independent phenomenon remains to be clarified. Moreover, whether retinal atrophy has a clinical (diagnostic and prognostic) value in these diseases remains unclear.

**Objective:**

To add light on the pathological significance and clinical value of retinal atrophy in patients with amyotrophic lateral sclerosis (ALS) and Kennedy’s disease (KD).

**Methods:**

Thirty-five ALS, thirty-seven KD, and forty-nine age-matched healthy controls (HC) were included in a one-year longitudinal study. Spectrum-domain optical coherence tomography (OCT) was performed at study entry (T0) and after 12 months (T1). Disease duration and functional rating scale (FRS) for ALS and KD patients were correlated to retinal thicknesses.

**Results:**

Compared to HC, peripapillary retinal nerve fiber layer (pRNFL) thickness was significantly thinner in both ALS (p = 0.034) and KD (p = 0.003). pRNFL was thinner in KD compared to ALS, but the difference was not significant. In KD, pRNFL atrophy significantly correlated with both disease severity (r = 0.296, p = 0.035) and disease duration (r = – 0.308, p = 0.013) while no significant correlation was found in ALS (disease severity: r = 0.147, p = 0.238; disease duration: r = – 0.093, p = 0.459). During the follow-up, pRNFL thickness remained stable in KD while significantly decreased in ALS (p = 0.043).

**Conclusions:**

Our study provides evidence of retinal atrophy in both ALS and KD and suggests that retinal thinning is a primary local phenomenon in motoneuron diseases. The clinical value of pRNFL atrophy in KD is worthy of further investigation.

## Introduction

Amyotrophic lateral sclerosis (ALS), the most common form of motor neuron disease (MND), and Kennedy’s disease (KD), an inherited MND that affects males, are two paradigmatic neurodegenerative diseases that strongly differ in clinical course. ALS is characterized by rapid neurodegeneration of upper and lower motor neurons, leading to death within 2–5 years following the diagnosis [1]. KD, caused by a genetic mutation of the androgen receptor gene on the X chromosome, is characterized by a slowly progressive loss of lower motor neurons (determining atrophy of limb and facial muscles), associated with other symptoms, such as sensory deficits, gynecomastia, and infertility [2].

Asymptomatic retinal nerve fiber layer (RNFL) thinning was reported to occur in ALS and was associated with disease progression, suggesting that other brain functional systems might be affected by the disease [3–5]. This observation, which constitutes a clinical/laboratory mismatch merits to be investigated given its pathogenic relevance. Moreover, no data are currently available on retina changes in other MND, particularly in KD.

Optical coherence tomography (OCT), a non-invasive imaging methodology that allows high-resolution analysis of retinal architecture and the measurement of its layers, is widely used to disclose early signs of neurodegeneration in brain inflammatory and neurodegenerative disorders. Indeed, thinning of the RNFL, the ganglion cell-inner plexiform layer (GCIPL), and the inner nuclear layer (INL) had been described in multiple sclerosis and dementia [6–14]. However, to what extent atrophy of retinal layers in brain disorders is secondary to the neurodegenerative processes occurring in the brain (i.e., trans-synaptic degeneration) or is primarily a local independent phenomenon remains to be clarified. Moreover, the clinical value of retinal atrophy remains obscure.

To add light on retinal pathology and its possible use for clinical purposes in MND, we designed a longitudinal OCT study aimed at analyzing and comparing retinal layer changes in two paradigmatic motoneuron diseases, ALS and KD, characterized by different pathogenetic mechanisms, severity and duration.

## Methods

### Study design and participants

Between April 2020 and September 2022, 35 ALS, 37 KD patients, and 49 healthy controls (HC) were consecutively enrolled in this study. ALS diagnosis was achieved according to the 2020 revised El Escorial criteria [15], and KD diagnosis was genetically acquired [16]. All patients and HC gave written informed consent. Exclusion criteria were (1) ophthalmologic pathologies (including iatrogenic optic neuropathy, diabetes, uncontrolled hypertension, glaucoma), (2) refractive errors (± 6 D), (3) inability to perform OCT examination (e.g. dropped head), (4) diagnosis of genetic ALS variant. All clinical and demographic data of patients and HC are summarized in Table 1.

**Table 1:** Baseline Demographics and Clinical Characteristics

	HC	ALS	Kennedy	HC vs ALS vs Kennedy p value	Bulbar onset ALS	Spinal onset ALS	Bulbar vs Spinal p value
Subjects (eyes) (%)	49 (98)	35 (66)	37 (71)	–	6 (10) (17%)	29 (56) (86%)	–
Age, years, mean (SD)	59.6 (10.9)	60.8 (8.1)	58.2 (11.0)	0.341^a^	60.5 (10.5)	60.8 (7.9)	0.930^b^
Male, n (%)	24 (49%)	21 (60%)	37 (100%)	HC vs ALS 0.158^cA^	1 (16.7%)	20 (69.0%)	**0.017**^**c**^
FRS, median (IQR)	–	40/48 (7.5)	41/56 (11.0)	–	5.5 (4.5–6.5)	6.0 (3.5–6.5)	0.766^d^
Disease duration, years, mean (SD)	–	2.10 (2.3)	13.7 (9.9)	< 0.001^b^	1.0 (1.3)	2.4 (2.4)	0.068^b^
On-going Riluzole therapy at baseline, n ALS patients (%)	–	29 (86%)	–	–	4 (66.7%)	25 (86.2%)	0.248^d^

*FRS* functional rating scale (ALSFRS-R for ALS: max score 48, SBMA-FRS for Kennedy: max score 56), *HC* healthy controls, *ALS* amyotrophic lateral sclerosis, *SD* standard deviation, *IQR* inter-quartile range

Bold indicates a statistically significant difference with a p value < 0.05

Significance testing

^a^one-way ANOVA

^b^2-tailed t test on means

^c^Chi-squared test

^d^Mann-Whitney test

^A^HC, and ALS vs Kennedy < 0.001

Cognitive and behavioral profile of all ALS patients included in the study was normal according to the Edinburgh Cognitive and Behavioral Amyotrophic Lateral Sclerosis screen (ECAS) which is routinely performed at our Center. In the large majority (> 80%) of patients the disease started in the spinal district which thus prevented us from conducting further statistical analysis clustering the patients by onset. On the other hand, statistical investigations by site of onset in KD patients are redundant since we assumed as disease onset the appearance of weakness in the limbs. Furthermore, according to the literature, cognitive impairment is minimal or absent in KD patients [17].

In ALS and KD patients OCT was performed at T0 (enrolment in the study) and after 12 months (T1), whereas in HC was done only at study entry. The Revised Amyotrophic Lateral Sclerosis Functional Rating Scale (ALSFRS-R) [18] and Spinal Bulbar Muscular Atrophy Functional Rating Scale (SBMA-FRS) score [19] were used as measures of disease severity, respectively in ALS and KD. For KD patients, disease duration was calculated since the onset of limb muscle weakness.

The study was approved by the Ethics Committee of University Hospital (Comitato Etico per la Sperimentazione, Azienda Ospedaliera Universitaria di Padova – Prot. N. AOP1619) and carried out in accordance with the Declaration of Helsinki.

### Optical coherence tomography

Spectral Domain (SD)-OCT (Spectralis; Heidelberg Engineering version 1.7.0.0) was performed by a single certified neurologist (AM) in accordance with the APOSTEL recommendations [20]. Data on global peripapillary RNFL (pRNFL) thickness (μm) were obtained using a 12-degree ring scan (corresponding to a 3.5 mm diameter) manually placed around the optic disc.

The peripapillary 3.5-mm ring scan was used to measure mean global peripapillary RNFL (pRNFL G) and mean sectorial peripapillary RNFL thickness, i.e. temporal (pRNFL T), temporal-superior (pRNFL TS), temporal-inferior (pRNFL TI), nasal (pRNFL N), nasal-superior (pRNFL NS), nasal-inferior (pRNFL NI). Moreover, mean thickness of the papillomacular bundle (pRNFL PMB) and ratio between the thickness of the nasal and that of the temporal sector (pRNFL N/T) were measured.

Data on the GCIPL, INL, outer plexiform-outer nuclear layer (OPNL), retinal pigment epithelium (RPE), inner retinal layer (IRL, including layers from RNFL to INL) volume (mm^3^) in the macular area were acquired using a macular volume scan centered on the fovea, and including a 6 mm ring area. Automated segmentation of OCT scans and quality control were performed. Scans violating international-consensus quality-control criteria (OSCAR-IB) [21] were excluded (n = 4 ALS and 3 KD patients excluded due to poor OCT quality; n = 35 ALS and 37 KD patients, of which respectively 66 and 71 eyes, entered the final analysis).

### Statistical analysis

Statistical analyses were performed using SPSS 22.0 (StataCorp LP, College Station, TX, USA). Normality in measurements was tested graphically and using Kolmogorov–Smirnov test. Nonparametric tests were used for non-normal or skewed data and parametric tests for normally distributed data. Respectively, median (interquartile range) and mean (± standard deviation) are shown. Differences between groups were analyzed using the chi-squared test for categorical variables, the independent 1-way ANOVA for parametric continuous variables, and the Mann–Whitney test for nonparametric continuous variables. Post-hoc comparisons were performed with a 2-tailed t tests corrected with Bonferroni procedure. Differences for segmented retinal layer thickness or volume data between groups were analyzed using generalized estimating equations (GEE) as recommended [20]; these were adjusted for intrasubject intereye correlations and repeated measurements, and employed an exchangeable correlation structure. To account even for potential effects of demographic and clinical variables we also corrected for age, gender, and z-score-transformed FRS. Relationship between RNFL thickness and the patients’ disease severity based on the correspondent Functional Rating Scale was assessed using the Spearman correlation, while Pearson correlation was used for the relationship between RNFL thickness and disease duration, expressed respectively in months (m) for ALS, and in years (y) for KD. A p value of 0.05 was accepted as statistically significant.

## Results

### Study populations

ALS, KD and HC did not differ in age (p = 0.341). HC and ALS did not differ in gender (p = 0.158), whereas an expected significant difference in gender (p < 0.001) and disease duration (p < 0.001) was found between ALS and KD, given respectively the X-linked inheritance and slower disease course of KD. Bulbar and spinal onset ALS were similar in age, ALSFRS-R score, disease duration, and on-going treatment. The majority (86%) of ALS patients were under Riluzole therapy at study enrollment (Table 1).

### Baseline comparisons of retinal layer thicknesses

**Fig. 1:**
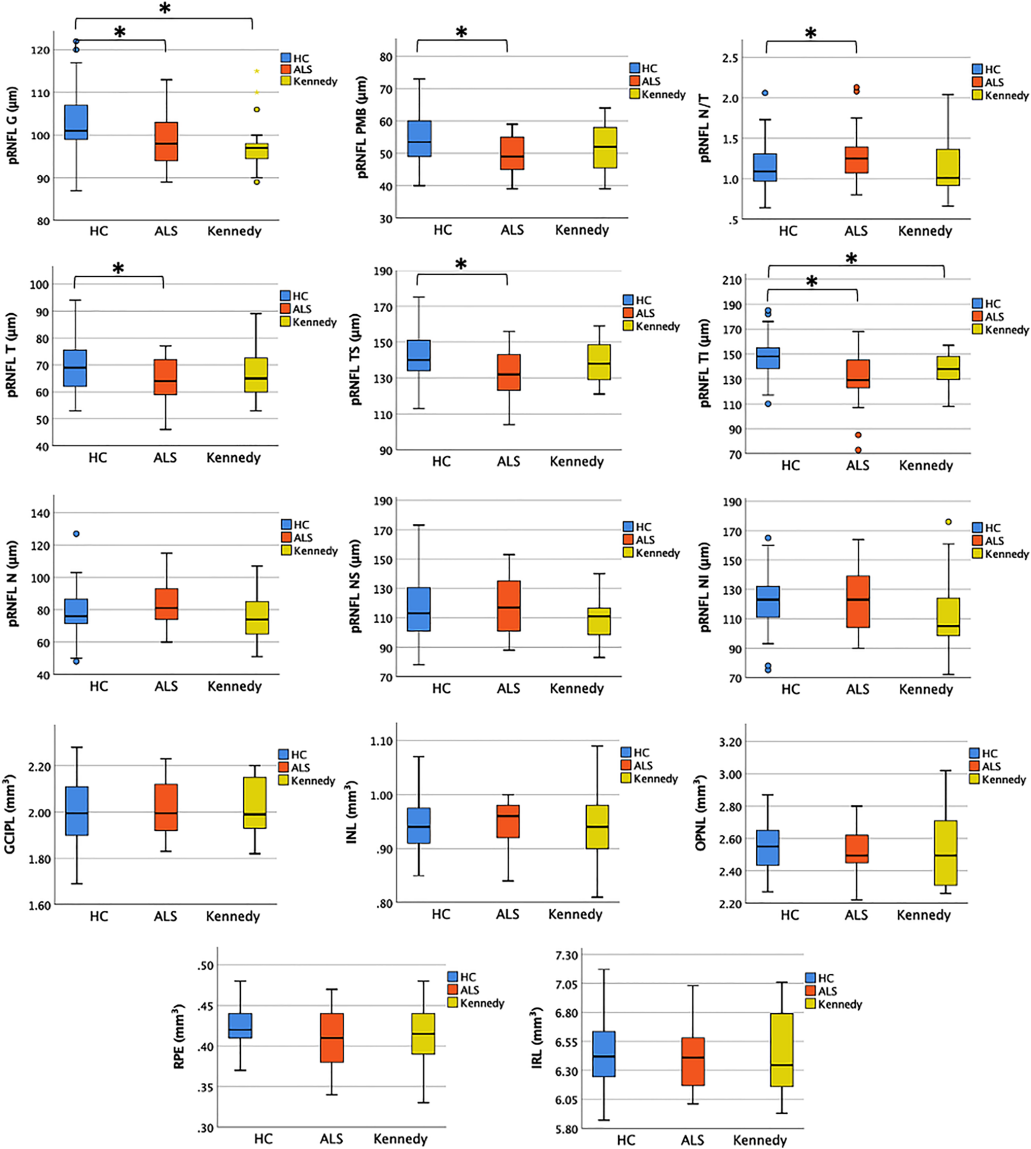
Bloxplots showing baseline comparisons between HC, ALS, and KD in peripapillary RNFL G, PMB, T/N ratio, and pRNFL sectors (thickness in μm), as well as macular GCIPL, INL, OPNL, RPE, IRL (volume in mm^3^). *indicates p < 0.05. pRNFL G thickness was significantly lower in both ALS (p = 0.034) and KD (p = 0.003) compared to HC. Sectorial analysis revealed that this significant thinning was localized in the whole temporal sectors (pRNFL N/T, T, TS, and TI) and PMB for ALS, and restricted to the TI sector in KD

At T0, pRNFL G thickness was significantly lower in both ALS (p = 0.034) and KD (p = 0.003) compared to HC. The same finding was observed when pRNFL G thickness was compared between KD and the male HC population (p = 0.012). pRNFL G was thinner in KD compared to ALS, but the difference did not reach significance. Interestingly, while ALS revealed the involvement of the whole temporal sectors (pRNFL N/T, T, TS, and TI) including the PMB, in KD retinal atrophy was significant only in the TI sector. All the other retina layers did not differ in volume between HC and the two MND groups (Table 2 and Fig. 1).

**Table 2:** Baseline retinal layers thickness and comparisons between groups

	HC	ALS	Kennedy	HC vs ALS	HC vs Kennedy	ALS vs Kennedy
				p value^a^	p value^a^	p value^a^
Thickness, μm, mean (SD)						
pRNFL G	103.13 (6.91)	99.76 (7.43)	97.79 (6.56)	0.034*	0.003*^,b^	0.353
pRNFL PMB	54.29 (6.66)	49.55 (5.69)	51.74 (8.02)	0.001*	0.161	0.275
pRNFL N/T	1.15 (0.27)	1.29 (0.31)	1.17 (0.37)	0.027*	0.807	0.223
pRNFL T	69.87 (9.32)	64.93 (8.06)	67.47 (10.00)	0.015*	0.333	0.337
pRNFL TS	142.18 (13.80)	131.24 (14.30)	138.32 (11.58)	0.001*	0.269	0.078
pRNFL TI	147.10 (15.84)	132.59 (22.30)	137.79 (13.59)	< 0.001*	0.022*	0.367
pRNFL N	78.37 (13.33)	81.76 (12.92)	75.53 (14.74)	0.250	0.424	0.129
pRNFL NS	116.46 (19.98)	118.52 (18.88)	110.32 (16.24)	0.637	0.222	0.127
pRNFL NI	122.57 (18.16)	122.07 (20.16)	113.79 (27.49)	0.904	0.202	0.235
Volume, mm^3^, mean (SD)						
GCIPL	2.013 (0.139)	2.023 (0.125)	2.014 (0.119)	0.742	0.982	0.800
INL	0.941 (0.046)	0.943 (0.050)	0.941 (0.066)	0.913	0.921	0.074
OPNL	2.547 (0.150)	2.505 (0.153)	2.531 (0.252)	0.214	0.800	0.680
RPE	0.422 (0.022)	0.409 (0.037)	0.410 (0.039)	0.072	0.063	0.873
IRL	6.428 (0.28)	6.419 (0.258)	6.394 (0.353)	0.573	0.906	0.776

*HC* healthy controls, *ALS* amyotrophic lateral sclerosis, *SD* standard deviation, *pRNFL* peri-papillary retinal nerve fiber layer, *G* global (average of all sectors), *PMB* papillo-macular bundle, *N/T* nasal/temporal ratio, *T* temporal sector, *TS* temporal superior sector, *TI* temporal inferior sector, *N* nasal sector, *NS* nasal superior sector, *NI* nasal inferior sector, *GCIPL* ganglion cell + inner plexiform layer, *INL* inner nuclear layer, *OPNL* outer plexiform + outer nuclear layer, *RPE* retinal pigment epithelium, *IRL* inner retinal layer (including layers from RNFL to INL)

ALS n (eyes) = 35 (66)

KD n (eyes) = 37 (71)

Significance testing

^*^p < 0.05

^a^Generalized estimation equation (GEE) model adjusted for inter-eye dependency

^b^GEE between KD and the only male HC: p = 0.012

**Fig. 2:**
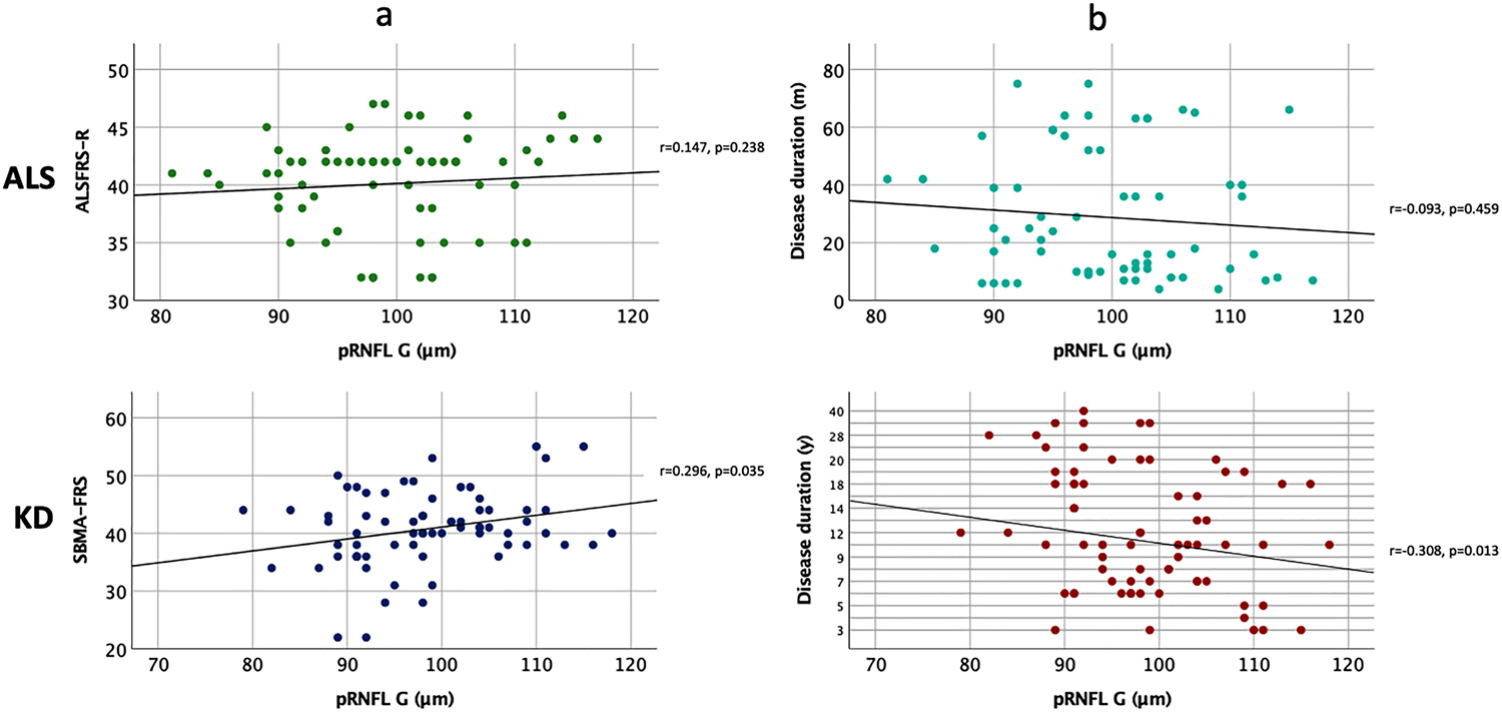
**a** Spearman correlation analysis between disease severity (based on ALSFRS-R and SBMA-FRS) and pRNFL G, respectively in ALS and KD patients. **b** Pearson correlation between pRNFL G and disease duration, expressed respectively in months (m) for ALS, and in years (y) for KD. r indicates either Spearman’s rho or Pearson r; p indicates significance. In KD, pRNFL G atrophy significantly correlated with both disease severity (r = 0.296, p = 0.035), and disease duration (r = – 0.308, p = 0.013)

In KD, pRNFL G atrophy significantly correlated with both disease severity (r = 0.296, p = 0.035), and disease duration (r = -0.308, p = 0.013), while in ALS, no significant correlation was found between pRNFL G thickness and both disease severity (r = 0.147, p = 0.238) or disease duration (r = -– 0.093, p = 0.459) (Fig. 2).

### Longitudinal analysis of retinal thickness changes

29/35 ALS and 35/37 KD patients completed the 1-year (T1) follow-up OCT examination. Causes of drop-out consisted in refusal of the second examination, neurological worsening, and death.

**Fig. 3:**
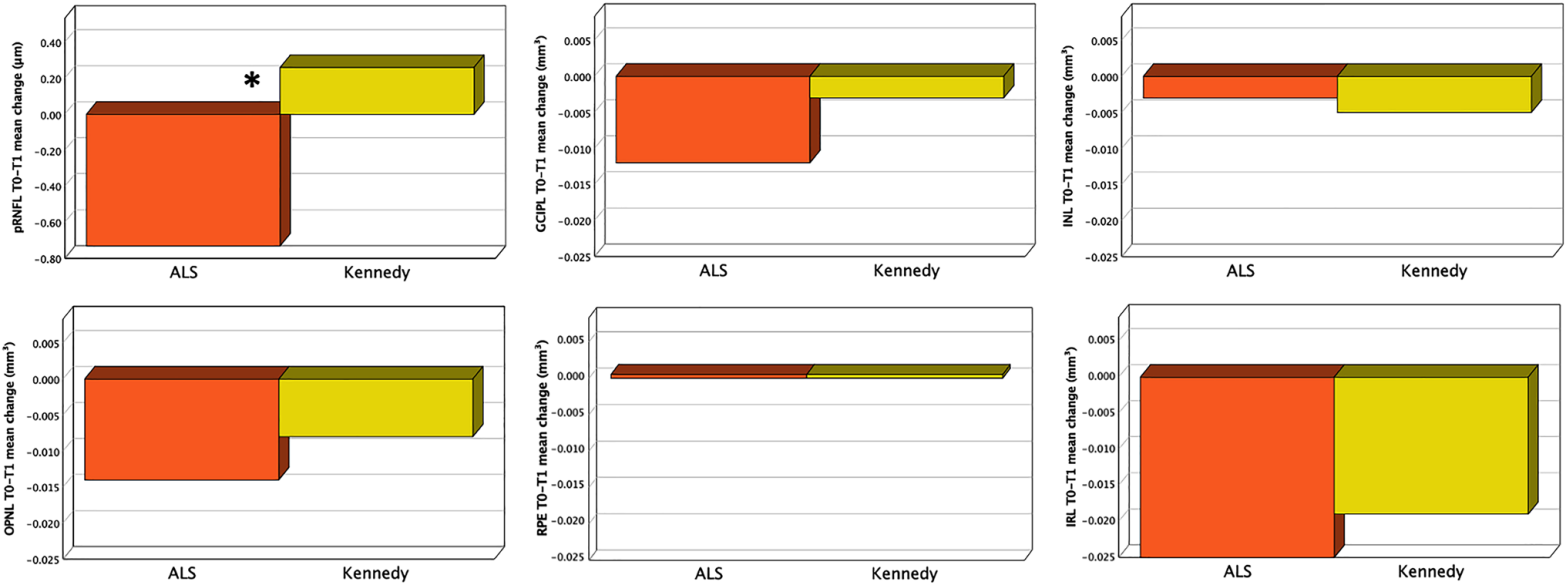
T0–T1 mean change in retinal pRNFL G thickness and GCIPL, INL, OPNL, RPE, IRL volume for ALS (orange bar) and KD (yellow bar). *indicates p < 0.05. pRNFL G thickness exhibited a stable annual trend in KD (from 97.85 ± 6.53 to 98.11 ± 7.66 μm; mean change of + 0.26 μm), while decreased in ALS (from 100.33 ± 7.95 to 99.60 ± 7.83 μm; mean change of – 0.73 μm), and the thickness change resulted significantly different between the two groups (p = 0.043)

From T0 to T1, pRNFL G thickness remained substantially stable in KD (from 97.85 ± 6.53 to 98.11 ± 7.66 μm; + 0.26%) but decreased in ALS (from 100.33 ± 7.95 to 99.60 ± 7.83 μm; -0.73%), and the thickness change was significantly different between the two groups (p = 0.043). No significant change in the volume of GCIPL, ONPL, RPE, IRL was observed between ALS and KD during the follow-up (Table 3 and Fig. 3).

**Table 3:** Comparison of change in retinal layers thickness between ALS vs Kennedy’s disease

	Motoneuron Disease	T0	T1	T0-T1 Percent Change, mean	Intra-group comparison	ALS vs Kennedy^b^
					T0–T1 p value^a^	
Thickness, μm, mean (SD)						
pRNFL G	ALS	100.33 (7.95)	99.60 (7.83)	– 0.73%	0.063	0.043*
	Kennedy	97.85 (6.53)	98.11 (7.66)	+ 0.26%	0.681	
Volume, mm^3^, mean (SD)						
GCIPL	ALS	2.029 (0.128)	2.017 (0.133)	– 0.59%	0.098	0.161
	Kennedy	2.014 (0.119)	2.011 (0.114)	– 0.15%	0.355	
INL	ALS	0.941 (0.050)	0.936 (0.049)	– 0.53%	0.264	0.604
	Kennedy	0.941 (0.066)	0.938 (0.058)	– 0.32%	0.480	
OPNL	ALS	2.512 (0.155)	2.498 (0.140)	– 0.56%	0.312	0.584
	Kennedy	2.531 (0.252)	2.523 (0.243)	– 0.32%	0.511	
RPE	ALS	0.411 (0.038)	0.411 (0.038)	0.00%	1.000	1.000
	Kennedy	0.409 (0.039)	0.409 (0.048)	0.00%	1.000	
IRL	ALS	6.412 (0.271)	6.387 (0.280)	– 0.39%	0.578	0.715
	Kennedy	6.419 (0.353)	6.400 (0.335)	– 0.30%	0.667	

*ALS* amyotrophic lateral sclerosis, *T0* baseline, *T1* twelve-month follow-up, *SD* standard deviation, *pRNFL G* peri-papillary retinal nerve fiber layer Global (average of all sectors), *GCIPL* ganglion cell + inner plexiform layer, *INL* inner nuclear layer, *OPNL* outer plexiform + outer nuclear layer, *RPE* retinal pigment epithelium, *IRL* inner retinal layer (including layers from RNFL to INL)

ALS n (eyes) = 29 (54)

KD n (eyes) = 35 (67)

^*^p < 0.05

^a^Generalised estimation equation (GEE) model adjusted for inter-eye dependency and repeated measurements

^b^Interaction time*group

## Discussion

Preliminary observations in ALS suggest that neurodegeneration is not confined to motor neurons but probably is a widespread pathological process that involves other functional systems of the central nervous system (CNS), such as the visual pathway [22]. Post-mortem studies disclosed neurodegeneration signs in the inner retinal layers of ALS patients, such as inclusion bodies and atrophy [23]. In vivo studies by OCT, although suggesting the involvement of retina, gave discordant results. While the majority of the studies agree on the significant thinning of RNFL compared to HC [3, 4, 22, 24], more contradictory data are available on INL atrophy [3, 22, 24–26]. Moreover, while some studies found an association between RNFL thinning and both motor function decline [4, 27] and disease duration [28], others failed [5, 26, 29]. Our study adds light on the structural retinal changes and their clinical correlates in ALS, also by comparison with the retinal changes observed in KD, up to date unexplored. Indeed, we observed that RNFL was atrophic in both ALS and KD, but with differences in thinning rate and involved sectors. Namely, ALS was associated with a more diffuse and faster pRNFL temporal atrophy compared to KD which was instead characterized by a limited pRNFL involvement, namely in the TI and NI sectors (even if the latter was characterized by a high variance which did not allow to reach the significance), whose thickness did not change during the follow-up. Although the evolution of retinal thinning in ALS seems to evolve in parallel with motoneuron degeneration, according to other studies [5, 26, 29], RNFL atrophy did not show a correlation with disease duration and severity. This observation, in line with pathological observations [23] seems to support the hypothesis of a primarily local neurodegenerative process occurring in the retina, that encourages further investigations.

To our knowledge, this is the first report describing retinal atrophy in KD patients. Besides lower motoneuron degeneration, KD is characterized by a complex clinical picture that may include sensory neuropathy, gynecomastia, autonomic and sexual dysfunctions. For the first time, we described a selective thinning of the TI sector of pRNFL in KD, and its association with disease severity and duration. These findings are particularly interesting since CNS seems to be spared in KD [30]. However, in a few studies on a very small number of patients subtle pathological changes in the grey matter or white matter atrophy were described by means of non-conventional MRI sequences (for a review see [31]). Most of the changes, however, were observed in the motor system, predominantly affecting the cortical-spinal tract [32] or the frontal lobe and cerebellum [33], although changes in the limbic lobe were also found. Although, MRI was not routinely performed on our patients (the exam was not included in the diagnostic workup), no patient in our cohort had clinical signs and symptoms of brain pathology. Thus, a primary slowly progressive neuronal degeneration (i.e., not detectable in a one-year follow-up), seems to occur in the retina of KD.

As above mentioned, to what extent RNFL atrophy is a primary local neurodegenerative phenomenon or rather the effect of trans-synaptic degeneration due to pathological changes in the visual pathway is a matter of debate. Indeed, the significant decrease in RNFL thickness observed in ALS in one-year follow-up, apparently not correlated with the clinical picture, seems to indicate that a local neurodegenerative process occurs in the retina of ALS patients, thus supporting the view that motor neurons are not the only neuronal populations affected in this disease. Moreover, our data in KD, a disease apparently not involving the CNS, further support the view that retina may be the site of primary neurodegenerative phenomena, that merit to be deeply investigated. Indeed, the retina is an extension of the CNS, and measurements of retina layers, especially RNFL thickness by means of OCT may provide important information on the neurodegenerative processes taking place in the brain. To what extent retinal thinning might reflect the aggressiveness and rapidity of the disease progression in ALS need to be explored in larger patient cohorts. However, by comparison, the absence of detectable retinal thinning in KD in our follow-up window indicates that retinal changes in KD proceed more slowly.

We are aware that the missing longitudinal trajectory of retinal changes in the HC group, accounting for natural aging-related thinning, may constitute a limitation of our study. However, we would like to stress the explorative nature of our study which was aimed at pointing out retinal differences between the two most important MND and their clinical correlates. Moreover, although quite heterogenous, the clinical phenotype of our ALS population was consistent with the epidemiological prevalence of spinal-onset ALS, compared to bulbar-onset, obtaining a representative sample of the sporadic ALS. To what extent the site of onset and the degree of behavioral impairment may influence the correlation with retinal findings is still unknown and deserves further investigation in larger patient cohorts.

In conclusion, our study in subjects with ALS or KD has added some new information about retinal neurodegeneration in MND. Taken together, our findings seem to suggest that retinal atrophy is a phenomenon at least partly disconnected from the degenerative process that characterizes MND.
